# Tranexamic acid and rosuvastatin in patients at risk of cardiovascular events after noncardiac surgery: a pilot of the POISE-3 randomized controlled trial

**DOI:** 10.1186/s40814-020-00643-9

**Published:** 2020-07-21

**Authors:** Maura Marcucci, Emmanuelle Duceppe, Yannick Le Manach, Clive Kearon, John W. Eikelboom, Kayla Pohl, Jessica Vincent, Saeed Darvish-Kazem, Sadeesh K. Srinathan, John D. D. Neary, Joel L. Parlow, Andrea Kurz, Peter L. Gross, Marko Mrkobrada, Kumar Balasubramanian, Daniel I. Sessler, P. J. Devereaux

**Affiliations:** 1 Department of Health Research Methods, Evidence, and Impact, McMaster University, Hamilton, ON Canada; 2 Department of Medicine, McMaster University, Hamilton, ON Canada; 3 Population Health Research Institute, Hamilton, ON Canada; 4 Department of Medicine, University of Montreal, Montreal, QC Canada; 5 Department of Anesthesia, McMaster University, Hamilton, ON Canada; 6 William Osler Health System, Brampton, ON Canada; 7 Department of Surgery, University of Manitoba, Winnipeg, MB Canada; 8 Department of Anesthesiology and Perioperative Medicine, Kingston General Hospital and Queen’s University, Kingston, ON Canada; 9 Department of Outcomes Research, Anesthesiology Institute, Cleveland Clinic, Cleveland, OH USA; 10 Department of Medicine, University of Western Ontario, London, ON Canada

**Keywords:** Tranexamic acid, Rosuvastatin, Noncardiac surgery, Pilot, Feasibility

## Abstract

**Background:**

Surgical bleeding is associated with postoperative cardiovascular complications. The efficacy and safety of tranexamic acid (TXA) in noncardiac surgery are still uncertain. Statins may prevent perioperative cardiovascular complications. We conducted a pilot to assess the feasibility of a perioperative trial of TXA and rosuvastatin.

**Methods:**

Using a factorial design, we randomized patients at cardiovascular risk undergoing noncardiac surgery to intravenous TXA (1 g at the start and end of surgery) or placebo, and oral rosuvastatin (40 mg before and 20 mg daily for 30 days after surgery) or placebo. Feasibility outcomes included recruitment rates, follow-up, and compliance to interventions. Clinical outcomes were secondarily explored.

**Results:**

After 3 months, we changed the design to a partial factorial due to the difficult recruitment of statin-naive patients. Over 6 months, 100 patients were randomized in the TXA trial (49 TXA, 51 placebo), 34 in the rosuvastatin trial (18 rosuvastatin, 16 placebo). Ninety-two percent (95% CI 80–98) of TXA and 86% (95% CI 74–94) of TXA-placebo patients received the 2 study doses. Thirty-three percent (95% CI 13–59) of rosuvastatin patients and 37% (95% CI 15–65) of rosuvastatin-placebo patients discontinued the study drug. A major cardiovascular complication occurred at 30 days in 1 TXA and 6 TXA-placebo patients, and 1 rosuvastatin and no rosuvastatin-placebo patients.

**Conclusions:**

Our pilot study supports the feasibility of a perioperative TXA trial in noncardiac surgery. Feasibility of a perioperative rosuvastatin trial is uncertain because of a high prevalence of statin use in the target population and concerns about compliance.

**Trial registration:**

ClinicalTrials.govNCT02546648.

## Background

Worldwide, more than 200 million noncardiac surgeries are performed annually [1, 2]. Although surgery has the potential to improve and prolong life, it can precipitate major complications. More than 15% of adults having surgery suffer a major complication (e.g., myocardial ischemic injury, bleeding, stroke) in the 30 days after surgery [3, 4]. Effective approaches to prevent these events are therefore urgently needed.

The pathogenesis of cardiovascular (CV) complications after noncardiac surgery is multifactorial [5, 6]. One mechanism involves perioperative bleeding, which is common and independently associated with postoperative myocardial infarction (MI) and stroke [7–9]. Tranexamic acid (TXA) is an anti-fibrinolytic agent that competitively inhibits the activation of plasminogen to plasmin [10]. TXA has the potential to reduce bleeding in noncardiac surgery, but its efficacy and safety in this clinical setting have not been established. There is a theoretical risk that TXA, by reducing thrombus breakdown, may cause CV events [11, 12]. However, by reducing bleeding, TXA may also prevent CV events. All noncardiac surgery studies on TXA to date are under-powered to investigate the effect on thromboembolic events and establish TXA overall benefit.

Experimental and observational evidence suggests that chronic preoperative statin therapy reduces postoperative CV complications and mortality [13–15]. The effect on statins on the endothelial function and inflammatory markers is rapid [16, 17] with a potential beneficial impact on clinical outcomes soon after the start of therapy, as demonstrated in patients undergoing percutaneous coronary intervention [18]. The existing evidence on statins to reduce perioperative CV events in vascular and non-vascular noncardiac surgery, coming from observational studies and small randomized controlled trials (RCTs) of limited methodological quality, is controversial and not definitive [13, 19–21].

There is a need of a large high-quality trial to address these questions. We did a pilot study, the Peri-Operative ISchemic Evaluation (POISE)-3 Pilot, with the primary objective of assessing the feasibility of a RCT of TXA versus placebo and of rosuvastatin versus placebo in patients undergoing noncardiac surgery.

## Methods

This report was prepared according to the CONSORT Extension to Pilot and Feasibility Trials guidelines (see Additional file 1 for the CONSORT checklist) [22].

### Study design

Using a factorial design, adult patients undergoing noncardiac surgery were randomized to receive either TXA or TXA-placebo and rosuvastatin or rosuvastatin-placebo. The pilot was conducted at the Hamilton General Hospital and at the Juravinski Hospital and Cancer Centre in Hamilton, Ontario, Canada.

The trial was approved by the local ethics committee. The pilot started as a full factorial RCT in which all patients had to be eligible and participate in both the TXA and rosuvastatin components. On July 6, 2015, the design was changed to a partial factorial in response to the initial low recruitment due to a high prevalence of statin use in potentially eligible patients.

### Participant eligibility and recruitment

Patients were eligible if they were having noncardiac surgery, ≥ 45 years of age, expected to have ≥ 1 night in the hospital after surgery, and a preoperative NT-pro-BNP measurement > 100 ng/mL. If NT-pro-BNP measurement was not available, the patient had to meet either of the following:

A) At least one of the following five criteria: history of coronary artery disease, history of peripheral vascular disease, history of stroke, undergoing major vascular surgery (i.e., vascular surgery except arteriovenous shunt, vein stripping procedures, and carotid endarterectomies); or
B) Any three of the following nine risk factors: undergoing major surgery defined as intraperitoneal, intrathoracic, retroperitoneal, or major orthopedic surgery; history of congestive heart failure; history of a transient ischemic attack; diabetes; hypertension; serum creatinine > 175 μmol/L; age > 70 years; history of smoking within 2 years of surgery; or undergoing emergent or urgent surgery.

Patients with any of the following were excluded: planned use of systemic TXA during surgery (topical use of non-study TXA was allowed), hypersensitivity or known allergy to TXA, creatinine clearance < 30 mL/min [Modification of Diet in Renal Disease (MDRD) Study equation], history of seizure disorder, history of venous thromboembolism, recent arterial thrombosis (≤ 30 days), recent subarachnoid hemorrhage (≤ 30 days), hematuria caused by diseases of the renal parenchyma, and no preoperative measurement of hemoglobin. After the change into the partial factorial design, the following became additional exclusion criteria only for the rosuvastatin trial: preoperative treatment with a statin or a non-statin lipid-lowering drug or ciclosporin during the 48 h before surgery, hypersensitivity or known allergy to rosuvastatin, and pre-disposed factors for myopathy or rhabdomyolysis (i.e., personal or family history of hereditary muscular disorders, previous history of muscle toxicity with the use of an HMG-CoA reductase inhibitor, severe hepatic impairment, hypothyroidism, or alcohol abuse). All participants provided written informed consent.

Recruitment took place between April and September 2015. Recruitment strategies included screening of patient list in the preoperative assessment clinic; daily surgical list in the operating room; patients in the preoperative holding area, on surgical wards and intensive care units; surgical admissions through the emergency department; and surgical consultations for ward patients. Anesthesia, surgery, and medicine services participated in the screening process.

### Randomization

Patients who provided informed consent underwent randomization before surgery by means of a 24-h computerized phone or web service that used variable-sized block randomization, stratified according to study center. Patients were assigned to receive TXA or matched placebo, and rosuvastatin or matched placebo, each in a 1:1 ratio. Patients, clinicians, data collectors, and outcome adjudicators were all unaware of the study group assignments.

### Interventions

Participants received a first dose (1 g) of TXA or matching placebo given intravenously over 10 min just after the induction of anesthesia and a second 1 g bolus dose over 10 min at the end of surgery. The dose given at the beginning of surgery was expected to provide therapeutic plasma concentrations for 120–180 min, with the dose given at the end of surgery providing additional therapeutic plasma concentrations for another 140–200 min [23]. The study dose regimen for TXA was chosen based on the review of the relevant literature. A meta-analysis of RCTs evaluating intravenous TXA in orthopedic surgery showed that dose regimens lower than 30 mg/kg are effective in reducing blood transfusion needs [24]. In the by-dose sub-analysis of a cohort study on 872,416 patients having total knee or hip arthroplasty in 510 hospitals, the 2000 mg dose seemed to have the best effectiveness and safety profile compared with ≤ 1000 mg or ≥ 3000 mg doses [25].

Patients received rosuvastatin or matching placebo at a dose of 40 mg orally before surgery (within 2 h before surgical incision). After surgery, the patients received 20 mg of rosuvastatin or matching placebo daily for 30 days, with the first postoperative dose given between 4 and 6 h after surgical closure.

### Study outcomes and follow-up

The feasibility outcomes included the rate of recruitment, completeness of follow-up at 30 days after surgery, and compliance to the study drug schedule. For TXA, we looked at the proportion of patients that received both and at least one of the 2 study drug doses. For rosuvastatin, we looked at the proportion of patients who took at least 80% of the study drug doses and the proportion of patients who discontinued the drug based on pre-specified definitions (see Additional file 2). We secondarily looked at clinical outcomes which are listed in Additional file 3.

All patients were contacted by phone at 30 days after randomization for outcome assessment. All relevant source documentation was collected. Clinical events were adjudicated by a committee of clinicians with expertise in perioperative outcomes, blinded to the treatment allocation.

### Sample size

A hundred patient convenience sample was used as this was believed to provide enough data to inform the feasibility outcomes.

### Statistical analyses

We analyzed patients in the treatment group to which they were allocated, according to the intention-to-treat principle. Descriptive statistics were used to summarize baseline and operative characteristics in tabular format by treatment. Compliance was reported as proportions with 95% CI.

The effect of the study interventions on the composite clinical outcomes was explored with time-to-event analyses and reported as hazard ratio (HR) and 95% CI derived from Cox proportional hazards models. The effect of TXA versus placebo on the hemoglobin change from preoperative to postoperative day 1, and from preoperative to the lowest postoperative value, was evaluated using the analysis of variance (ANOVA). We also performed a subgroup analysis according to the use of topical TXA and tested the effect modification including the interaction term in an ANCOVA model.

Statistical significance was inferred for all comparisons if the computed 2-sided *p* value was < 0.05. All analyses were performed with the use of SAS software, version 9.4.

## Results

### Patients

**Fig. 1:**
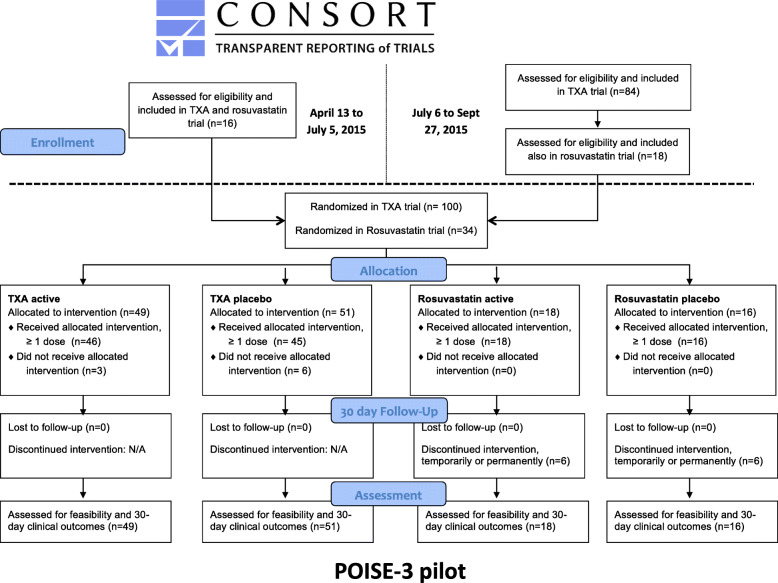
The POISE-3 pilot flow diagram. The figure shows the study flow diagram based on the CONSORT Extension to Pilot and Feasibility Trials guidelines [22]

The trial flow diagram with recruitment data for the two study phases is presented in Fig. 1. Overall, over 6 months, 100 patients were enrolled in the TXA factorial; 49 patients were randomized to TXA, 51 to TXA-placebo. Thirty-four of these patients were also enrolled in the rosuvastatin trial (18 randomized to rosuvastatin and 16 to rosuvastatin-placebo).

Characteristics at baseline of the enrolled patients are presented in Table 1, Table 2, and Additional file 4, separately for the two trials and their arms. In the TXA trial, the mean age was 69.7 years [standard deviation (SD) 8.6]; 54.0% were men. In the rosuvastatin trial, the mean age was 68.4 years (SD 9.7); 52.9% were men. There was a history of coronary artery disease in 29.0% of patients in the TXA trial and 8.8% of patients in the rosuvastatin trial. The most common type of surgery was orthopedic surgery in both factorials, accounting for 45.0% of patients in the TXA and 52.9% in the rosuvastatin trial. Overall, 11 patients (11.0%) in the TXA trial, and 4 (11.8%) in the rosuvastatin trial, were on oral anticoagulant therapy at baseline; none of them received the oral anticoagulant therapy in the 24 h preceding surgery (Additional file 4).

**Table 1:** Patient baseline characteristics

**Characteristic**	**TXA (*N*** **= 49)**	**TXA placebo (*N*** **= 51)**	**Rosuvastatin (*N*** **= 18)**	**Rosuvastatin placebo (*N*** **= 16)**
Mean age ± SD, years	70.0 ± 8.2	69.5 ± 8.9	68.4 ± 9.1	68.4 ± 10.6
Male sex, *n* (%)	26 (53.1)	28 (54.9)	12 (66.7)	6 (37.5)
Eligibility criteria met, *n* (%)				
Age ≥ 45 years	49 (100)	51 (100)	18 (100)	16 (100)
Preoperative NT-pro-BNP > 100 ng/mL	5 (10.2)	5 (9.8)	1 (5.6)	6 (37.5)
Patients fulfilling at least one or more of the following 5 criteria	48 (98.0)	50 (98.0)	18 (100)	14 (87.5)
1. History of coronary disease	15 (30.6)	14 (27.5)	2 (11.1)	1 (6.3)
2. History of peripheral vascular disease	7 (14.3)	8 (15.7)	5 (27.8)	3 (18.8)
3. History of stroke	2 (4.1)	1 (2.0)	0 (0.0)	1 (6.3)
4. Undergoing major vascular surgery	2 (4.1)	3 (5.9)	1 (5.6)	1 (6.3)
5. Patients fulfilling any 3 of the following risk factors	35 (71.4)	35 (68.6)	13 (72.2)	12 (75.0)
Undergoing major surgery	29 (59.2)	31 (60.8)	14 (77.8)	12 (75.0)
History of congestive heart failure	1 (2.0)	3 (5.9)	1 (5.6)	0 (0.0)
History of transient ischemic attack	2 (4.1)	4 (7.8)	0 (0.0)	0 (0.0)
Diabetes and currently taking an oral hypoglycemic	19 (38.8)	16 (31.4)	3 (16.7)	4 (25.0)
Age ≥ 70 years	28 (57.1)	31 (60.8)	11 (61.1)	8 (50.0)
Hypertension	41 (83.7)	46 (90.2)	15 (83.3)	14 (87.5)
Serum creatinine > 175 μmol/L	0 (0.0)	0 (0.0)	0 (0.0)	0 (0.0)
History of smoking within 2 years of surgery	12 (24.5)	14 (27.5)	6 (33.3)	5 (31.3)
Undergoing emergent/urgent surgery	0 (0.0)	0 (0.0)	0 (0.0)	0 (0.0)
Mean preoperative hemoglobin ± SD, g/L	134.7 ± 15.7	130.0 ± 14.1	135.3 ± 17.5	137.4 ± 13.3
Serum creatinine (μmol/L)	88.3 ± 29.2	90.1 ± 27.6	77.7 ± 16.2	80.4 ± 23.9
Mean time from randomization to start of surgery ± SD^a^, hours	29.8 ± 18.6	32.2 ± 22.0	29.7 ± 19.9	31.4 ± 21.8
Time from randomization to start of surgery, *n* (%)				
≤ 24 h	21 (42.9)	24 (47.1)	9 (50.0)	8 (50.0)
24–48 h	23 (46.9)	18 (35.3)	6 (33.3)	6 (37.5)
> 48 h^b^	5 (10.2)	8 (16.0)	3 (16.7)	2 (12.5)

*TXA* tranexamic acid, *SD* standard deviation

^a^Percentages are reported among the total of patients, i.e., the denominator includes also patients in which NT-proBNP was not measured

^b^After excluding from the calculation a patient for whom surgery was canceled after randomization and postponed to 64 days later

**Table 2:** Intraoperative characteristics

**Characteristic**	**TXA (*N*** **= 49)**	**TXA placebo (*N*** **= 51)**	**Rosuvastatin (*N*** **= 18)**	**Rosuvastatin placebo (*N*** **= 16)**
Type of surgery, *n* (%)				
Major vascular surgery^a^	8 (16.3)	7 (13.7)	4 (22.2)	2 (12.5)
Other vascular surgery	5 (10.2)	2 (3.9)	0 (0.0)	1 (6.3)
Thoracic surgery	0 (0.0)	1 (2.0)	0 (0.0)	0 (0.0)
Major orthopedic surgery^b^	21 (42.9)	21 (41.2)	9 (50.0)	8 (50.0)
Other orthopedic surgery	3 (6.1)	0 (0.0)	0 (0.0)	1 (6.3)
Urology or gynecology surgery	2 (4.1)	3 (6.0)	1 (5.6)	0 (0.0)
Major general surgery^c^	5 (10.2)	6 (11.8)	2 (11.1)	2 (12.5)
Spinal surgery	4 (8.2)	9 (17.6)	2 (11.1)	2 (12.5)
Low-risk surgeries^d^	1 (2.0)	2 (3.9)	0 (0.0)	0 (0.0)
Type of anesthesia, *n* (%)				
General	28 (57.1)	29 (56.9)	8 (44.4)	12 (75.0)
Spinal	22 (44.9)	25 (49.0)	11 (61.1)	6 (37.5)
Epidural	6 (12.2)	4 (7.8)	2 (11.1)	2 (12.5)
Plexus/nerve block	0 (0.0)	2 (3.9)	1 (5.6)	0 (0.0)
Sedation	5 (10.2)	4 (7.8)	1 (5.6)	1 (6.3)
Intraoperative administration of non-study topical TXA	22 (44.9)	22 (43.1)	9 (50.0)	9 (56.3)
Mean total dose ± SD, g	3.0 ± 0.0	3.0 ± 0.2	3.0 ± 0.0	3.0 ± 0.0

*TXA* tranexamic acid, *SD* standard deviation

^a^Major vascular surgery included thoracic aorta reconstructive vascular surgery, aorto-iliac reconstructive vascular surgery, peripheral vascular reconstruction without aortic cross-clamping, and extracranial cerebrovascular surgery

^b^Major orthopedic surgery included major hip or pelvic surgery, internal fixation of femur, knee arthroplasty, above-knee amputation, and lower leg amputation

^c^Major general surgery included complex visceral resection; partial or total colectomy, or stomach surgery; small bowel resection; and major head and neck resection for non-thyroid tumor

^d^Low-risk surgery included any other surgery not included above or any of the following surgeries: parathyroid, thyroid, breast, hernia, local anorectal procedure, oophorectomy, salpingectomy, endometrial ablation, peripheral nerve surgery, ophthalmology, vertebral disc surgery, hand surgery, metatarsal resection, cosmetic surgery, arterio-venous access surgery for dialysis

### Study outcomes

#### Feasibility outcomes

The overall recruitment rate was 5.8 patients per month (95% CI 3.3–9.4) for both the TXA and rosuvastatin trials during the initial full factorial phase of the trial. After the change to the partial factorial design, the overall recruitment rate was 32.7 patients per month (95% CI 26.1–40.5) for the TXA trial and 7.0 patients per month (95% CI 4.1–11.1) for the rosuvastatin trial, with both centers recruiting at the same rate. All patients (100%) completed the 30-day follow-up.

Table 3 shows the compliance to the study drug schedule in the TXA trial; 89.0% of the patients received both doses of the study drug.

**Table 3:** Feasibility outcome: compliance in TXA trial

	**All (*N*** **= 100)**	**TXA (*N*** **= 49)**	**Placebo (*N*** **= 51)**
Patients who received both doses of study drug, *n* (%; 95% CI)	89 (89.0; 81.2–94.4)	45 (91.8; 80.4–97.7)	44 (86.3; 73.7–94.3)
Patients who received only one dose of study drug, *n* (%; 95% CI)	2 (2.0; 0.2–7.0)	1 (2.0; < 0.1–10.8)	1 (2.0; < 0.1–10.4)
Patients who received no dose of study drug, *n* (%; 95% CI)	9 (9.0; 4.2–16.4)	3 (6.1; 1.3–16.9)	6 (11.8; 4.4–23.9)

*TXA* tranexamic acid, *CI* confidence interval.

In the rosuvastatin trial, 25 (73.5%, 95% CI 55.6–87.1) of patients took at least 80% of the study drug doses: 13 (72.2%, 95% CI 46.5–90.3) in the rosuvastatin and 12 (75%, 95% CI 47.6–92.7) in the placebo trial. According to the study definitions (see Additional file 2), 12 patients (35.3%, 95% CI 19.7–53.5) discontinued the study drug; 8 of them never resumed the drug by the 30th day after surgery [23.5%, 95% CI 10.7–41.2; 5 in the rosuvastatin (27.8%) and 3 in the placebo group (18.8%)]. The reason for stopping the study drug was “patient forgot/refusal” in 6 of the 8 patients; study medications were lost by 1 patient and stopped in another patient because it was discovered that the patient was already on statin therapy.

#### Clinical outcomes

In the TXA trial, patients stayed in the hospital for a median of 3 days [first (Q1) and third quartile (Q3) 2.0, 5.0 days] and in the intensive care unit/cardiac intensive care unit (ICU/CICU) for a mean of 0.5 days (SD 3.0 days); there was no statistically significant difference between the study groups. In the rosuvastatin trial, patients stayed in the hospital for a median of 2.5 days [first (Q1) and third quartile (Q3) 2.0, 5.0 days] and in the ICU/CICU for a mean of 0 days (SD 0.2 days); there was no statistically significant difference between the study groups.

The mean change in hemoglobin levels did not differ between the TXA and TXA-placebo group (see Additional file 5). Non-study topical TXA was administered to 44 patients, 45% and 43% of the patients in the TXA and in the placebo group, respectively (Table 2). All but 1 patient (placebo group, thoracic surgery) receiving topical TXA underwent orthopedic surgery. The use of topical TXA did not modify the effect of the study TXA on the change in preoperative hemoglobin to the hemoglobin on postoperative day 1 (*p* for interaction 0.24), or to the lowest postoperative hemoglobin (*p* for interaction 0.49).

Other clinical outcomes in the TXA trial are presented in Table 4. The composite outcome of vascular mortality, non-fatal myocardial infarction, non-fatal stroke, non-fatal cardiac arrest, non-fatal pulmonary embolism, non-fatal deep vein thrombosis, non-fatal life-threatening bleeding, and non-fatal major bleeding occurred in 1 patient randomized to TXA and 6 patients randomized to placebo (HR, 0.16; 95% CI 0.02–1.34). No serious adverse event (SAE) was reported.

**Table 4:** Clinical outcomes in TXA trial

**Outcomes**	**TXA (*N*** **= 49)**	**Placebo (*N*** **= 51)**	**HR (95% CI)**
Composite outcome within 30 days after surgery^a^, *n* (%)	1 (2.0%)	6 (11.8%)	0.16 (0.02–1.34)
Vascular mortality, *n* (%)	0 (0.0)	1 (2.0)^b^	
Non-fatal myocardial infarction, *n* (%)	1 (2.0%)	3 (5.9%)	
Stroke, *n* (%)	0 (0.0)	0 (0.0)	
Pulmonary embolism, *n* (%)	0 (0.0)	0 (0.0)	
Deep vein thrombosis, *n* (%)	0 (0.0)	1 (2.0)	
Life-threatening bleeding, *n* (%)	0 (0.0)	2 (3.9)	
Non-fatal major bleeding, *n* (%)	0 (0.0)	1 (2.0)	
All-cause mortality, *n* (%)	0 (0.0)	1 (2.0)	–^c^
Myocardial infarction, *n* (%)	1 (2.0%)	4 (7.8%)	0.23 (0.03–2.07)
MINS, *n* (%)	0 (0.0%)	6 (11.8%)	–^c^
Cardiac revascularization, *n* (%)	0 (0.0)	1 (2.0)	–^c^
Postoperative infection, *n* (%)	6 (12.2)	7 (13.7)	0.77 (0.26–2.30)
Postoperative sepsis, *n* (%)	1 (2.0)	4 (7.8)	0.23 (0.03–2.04)
Congestive heart failure, *n* (%)	1 (2.0)	0 (0.0)	–^c^
Re-hospitalization for vascular reason, *n* (%)	0 (0.0)	1 (2.0)	–^c^
Seizure, *n* (%)	0 (0.0)	0 (0.0)	–^c^

*TXA* tranexamic acid, *HR* hazard ratio, *CI* confidence interval, *MINS* myocardial injury after noncardiac surgery

^a^Composite outcome of vascular mortality, non-fatal myocardial infarction, non-fatal stroke, non-fatal cardiac arrest, non-fatal pulmonary embolism, non-fatal deep vein thrombosis, non-fatal life-threatening bleeding, and non-fatal major bleeding

^b^Due to myocardial infarction

^c^Not determinable

Clinical outcomes in the rosuvastatin trial are presented in Table 5. There was only 1 major CV event in the rosuvastatin trial. No patient complained about myalgia or other myopathies; no SAE was reported.

**Table 5:** Clinical outcomes in rosuvastatin trial

**Outcomes**	**Rosuvastatin (*N*** **= 18)**	**Placebo (*N*** **= 16)**	**HR (95% CI)**
Composite of all-cause mortality or non-fatal myocardial infarction within 30 days after surgery, *n* (%)	1 (5.6)	0 (0.0)	–^a^
All-cause mortality, *n* (%)	0 (0.0)	0 (0.0)	
Non-fatal myocardial infarction, *n* (%)	1 (5.6%)	0 (0.0%)	
Myocardial infarction, *n* (%)	1 (5.6%)	0 (0.0%)	–^a^
MINS, *n* (%)	2 (11.1)	2 (12.5%)	0.92 (0.13–6.64)
Cardiac revascularization, *n* (%)	0 (0.0)	0 (0.0)	–^a^
Venous thromboembolism, *n* (%)	0 (0.0)	0 (0.0)	–^a^
Postoperative infection, *n* (%)	4 (22.2)	1 (6.3)	3.36 (0.37–30.4)
Postoperative sepsis, *n* (%)	2 (11.1)	0 (0.0)	–^a^
Congestive heart failure, *n* (%)	0 (0.0)	0 (0.0)	–^a^
Re-hospitalization for vascular reason, *n* (%)	0 (0.0)	0 (0.0)	–^a^
Statin-induced myopathy, *n* (%)	0 (0.0)	0 (0.0)	–^a^

*TXA* tranexamic acid, *HR* hazard ratio, *CI* confidence interval, *MINS* myocardial injury after noncardiac surgery

^a^Not determinable

## Discussion

The present pilot confirmed the feasibility of a perioperative trial on TXA in patients undergoing noncardiac surgery. In particular, we showed the feasibility of recruiting eligible patients. Overall, TXA drug compliance was good with 89% of patients receiving both doses of the study drug. Moreover, we can optimistically expect that the compliance rate will increase in the main trial, as more patients are included and the participating centers become more familiar with the study procedures.

In our pilot, the effect of intravenous TXA on the composite outcome including thrombotic and bleeding events was in the direction of no harm and potential benefit. In particular, fatal and non-fatal bleeding, but also MI and myocardial injury after noncardiac surgery (MINS), were more frequent in the placebo than in the TXA group (Table 4). Although only exploratory, these findings are encouraging. TXA was not associated with a significant reduction in the hemoglobin drop after surgery, compared with placebo. Previous (small) RCTs and systematic reviews in noncardiac surgery demonstrated a favorable effect of TXA on blood loss and/or transfusion rate [26–30]. However, only a minority of the existing studies looked at the effect on perioperative hemoglobin change, which could explain at least part of the difference between our and others’ findings. Even if the hemoglobin drop has physiologic and clinical relevance, there are several factors that can affect it, such as dilution due to iv fluid therapy and preoperative or intraoperative blood transfusions. This might make the hemoglobin drop not the most sensitive outcome to test the effect of an antifibrinolytic drug expected to reduce bleeding. Then, the different case mix in our study compared with other studies might also explain our findings. Previous RCTs on TXA in noncardiac surgery looked at one specific patient population, and largely most of them were conducted in orthopedic patients. Conversely, 42% of our patients underwent major joint surgery, but we also included other types of surgery, which might have added heterogeneity and further reduced our power. Thus, our pilot helped emphasize the need for a larger trial to test the effect of TXA on bleeding in different types of noncardiac surgery and using outcome definitions that cover different aspects of the clinical relevance and severity of a bleeding event (i.e., extent of the hemoglobin drop; need for interventions like transfusion, surgical intervention, or hemodynamic support; and fatality).

Finally, even if conducted in only 2 centers, our pilot also informed on the current practice about TXA use. Patients in whom the use of systemic TXA during surgery was already planned were not eligible for the study, while we allowed surgeons to use non-study TXA topically based on their usual practice. Almost all patients undergoing joint arthroplasty in our Hamilton centers received topical TXA in addition to the study drug. Although we expect some heterogeneity across countries and centers in the larger trial, this finding suggests that allowing for use of topical TXA might increase the acceptance from physicians and feasibility of the trial. Recent studies and systematic reviews in joint arthroplasty suggested that the combination of intravenous and topical TXA might be more effective in reducing blood loss and transfusion rates [31–34]. We will explore whether the topical use of non-study TXA is an effect modifier of the efficacy of the study TXA in the main trial. In our pilot, we did not show any interaction with the effect on the hemoglobin drop, but unfortunately, all patients undergoing major orthopedic surgery were treated with topical TXA. Hence, we could not test the interaction within major orthopedic surgery; also, we could not separate the possible effect modification due to the topical use from the possible effect modification related to the type of surgery. On the other side, the pilot was preliminarily reassuring with regard to the safety of our decision. None of the major CV events (Table 4) occurred in patients receiving both study (intravenous) and non-study (topical) TXA.

In the POISE-3 pilot trial, according to a partial factorial design, 34 patients were also randomized to receive rosuvastatin 20 mg or matching placebo, with a first dose before surgery and then daily for 30 days. We had anticipated that the effect of statin in perioperative CV complications would be better studied in patients who are not chronically on statin, and designed the study accordingly. However, we experienced substantial difficulty in recruiting patients naive to statin who, at the same time, would meet our core inclusion criteria for increased CV risk. Also, among those eventually enrolled, more than 30% of patients missed some doses of the study drug, and 24% of patients (28% in the rosuvastatin group) discontinued it permanently before the end of the 30 days. No patient experienced statin-induced myopathy or any serious adverse effect as a possible explanation for the withdrawal from the study medication. These percentages of non-compliance met our prespecified threshold for considering the main study non-feasible unless protocol modifications were to be implemented (Additional file 2). However, the challenges with recruiting high-risk patients, not already on statin, would remain a main challenge.

## Conclusions

Our pilot study demonstrated the feasibility of a perioperative TXA trial in noncardiac surgery. Moreover, it did not show any signal of harm of TXA also in a setting and population at high risk of CV events. Based on the findings of this pilot, we launched the main POISE-3 trial, which will evaluate the efficacy of TXA in reducing clinically important bleeding (superiority hypothesis) and at the same time its safety on major arterial and venous thrombotic events (non-inferiority hypothesis), in 10,000 patients 45 years of age or older, with or at risk of CV disease, undergoing different types of noncardiac surgery, in more than 25 countries worldwide. On the other side, our pilot suggested that a trial on statin in the perioperative setting in patients at an increased baseline risk of CV complications, but naive to statin therapy, will likely encounter feasibility issues in terms of patient recruitment and compliance to the study protocol.

## Supplementary information

**Additional File 1.** CONSORT checklist for POISE-3 Pilot manuscript

**Additional File 2.** Definitions of postoperative drug discontinuation in the rosuvastatin trial.

**Additional File 3.** List of prespecfied clinical outcomes for the tranexamic acid versus placebo and the rosuvastatin versus placebo trials.

**Additional File 4.** Additional table. Patient characteristics at baseline: medications.

**Additional File 5.** Additional Table. Effect of TXA on hemoglobin change from preoperative to post-operative day 1, and from preoperative to the lowest postoperative level.

## Data Availability

Data can be provided upon request to Dr. PJ Devereaux at PJ.Devereaux@phri.ca.

## Abbreviations

CV: Cardiovascular
ICU/CICU: Intensive care unit/cardiac intensive care unit
MI: Myocardial infarction
MINS: Myocardial injury after noncardiac surgery
RCT: Randomized controlled trial
SAE: Serious adverse event
TXA: Tranexamic acid
