# Supplementary material for: Tranexamic acid and rosuvastatin in patients at risk of cardiovascular events after noncardiac surgery: a pilot of the POISE-3 randomized controlled trial
Source: Pilot Feasibility Stud. 2020 Jul 21;6:104. doi: 10.1186/s40814-020-00643-9 (PMC7372857; doi:10.1186/s40814-020-00643-9)
Supplement: Supplementary file 2 — Additional File 2. Definitions of postoperative drug discontinuation in the rosuvastatin trial. [file 40814_2020_643_MOESM2_ESM.doc]

**Definitions of postoperative drug discontinuation in the rosuvastatin trial**

**Postoperative cumulative discontinuation:** a patient met the definition of postoperative cumulative discontinuation when the patient missed at least 2 doses (cumulatively, not necessarily consecutively) during the first 10 postoperative days (first dose after surgery, on the day of surgery, included), or at least 3 doses (cumulatively, not necessarily consecutively) from postoperative day 10 to postoperative day 30, regardless of resumption after the last missed dose.

**Postoperative permanent discontinuation**: a postoperative cumulative discontinuation with no resumption at 30 days after randomization.

We prespecified the following criteria to evaluate the feasibility of the trial upon the proportion of postoperative cumulative discontinuation: 1) a ≤ 20% drug discontinuation proportion would suggest to continue without modification; 2) a 21-39% drug discontinuation proportion would suggest to continue with protocol modifications; 3) a ≥40% of drug discontinuation would suggest to stop and declare the main study non feasible. The emphasis on the role of drug discontinuation (even when not permanent) was dictated by the evidence from *in vitro* and clinical studies that the positive vascular effects of statins have a rapid offset after drug withdrawal.

References:

Gertz K, Laufs U, Lindauer U, et al. Withdrawal of statin treatment abrogates stroke protection in mice. Stroke 2003;34:551-7.

Taneva E, Borucki K, Wiens L, et al. Early effects on endothelial function of atorvastatin 40 mg twice daily and its withdrawal. Am J Cardiol 2006;97:1002-6.

Spencer FA, Fonarow GC, Frederick PD, et al. Early withdrawal of statin therapy in patients with non-ST-segment elevation myocardial infarction: national registry of myocardial infarction. Arch Intern Med 2004;164:2162-8.
