# Supplementary material for: Tranexamic acid and rosuvastatin in patients at risk of cardiovascular events after noncardiac surgery: a pilot of the POISE-3 randomized controlled trial
Source: Pilot Feasibility Stud. 2020 Jul 21;6:104. doi: 10.1186/s40814-020-00643-9 (PMC7372857; doi:10.1186/s40814-020-00643-9)
Supplement: Supplementary file 3 — Additional File 3. List of prespecfied clinical outcomes for the tranexamic acid versus placebo and the rosuvastatin versus placebo trials. [file 40814_2020_643_MOESM3_ESM.doc]

**List of prespecfied clinical outcomes for the tranexamic acid versus placebo and the rosuvastatin versus placebo trials**

| **Tranexamic acid versus placebo trial: clinical outcomes** | **Rosuvastatin versus placebo trial: clinical outcomes** |
| --- | --- |
| change in preoperative hemoglobin to day 1 after surgery | - |
| change in preoperative hemoglobin to the lowest level of hemoglobin reached postoperatively |  |
| composite of vascular mortality and nonfatal myocardial injury after noncardiac surgery (MINS), stroke, cardiac arrest, pulmonary embolism, deep venous thrombosis, life threatening bleeding, and major bleeding; and individual components of the composite | composite of all-cause mortality and nonfatal MI; and individual components of the composite |
|  | myocardial injury after noncardiac surgery (MINS)* |
|  | stroke |
|  | cardiac arrest |
|  | venous thromboembolism |
| cardiac revascularization procedure | cardiac revascularization procedure |
| transfusion | transfusion |
| clinically important hypotension | clinically important hypotension |
| postoperative infection | postoperative infection |
| length of hospital stay | length of hospital stay |
| length of intensive care unit/cardiac care unit (ICU/CCU) stay | length of intensive care unit/cardiac care unit (ICU/CCU) stay |
| re-hospitalization for vascular reasons | re-hospitalization for vascular reasons |
| seizure | statin-induced myopathy |

*MINS was defined as any elevated troponin (higher than the local lab threshold) judged to be due to myocardial ischemia (i.e. without evidence of a non-ischemic etiology, e.g. chronic elevation, pulmonary embolism, sepsis, cardioversion, others) that occurred within the first 30 days after surgery
