# Supplementary material for: Tranexamic acid and rosuvastatin in patients at risk of cardiovascular events after noncardiac surgery: a pilot of the POISE-3 randomized controlled trial
Source: Pilot Feasibility Stud. 2020 Jul 21;6:104. doi: 10.1186/s40814-020-00643-9 (PMC7372857; doi:10.1186/s40814-020-00643-9)
Supplement: Supplementary file 4 — Additional File 4. Additional table. Patient characteristics at baseline: medications. [file 40814_2020_643_MOESM4_ESM.doc]

**Additional table**. Patient characteristics at baseline: medications

| **Characteristic** | **TXA**  **(N=49)** | **TXA placebo (N=51)** | **Rosuvastatin (N=18)** | **Rosuvastatin placebo (N=16)** |
| --- | --- | --- | --- | --- |
| **Medications taken <7 days to >24 hr before surgery, n (%)** |  |  |  |  |
| Non-study Statin | 33 (67.3) | 30 (58.8) | 1 (5.6)* | 0 (0.0) |
| Therapeutic dose warfarin | 1 (2.0) | 1 (2.0) | 0 (0.0) | 2 (12.5) |
| Therapeutic dose direct thrombin/factor Xa inhibitor | 5 (10.2) | 4 (7.8) | 2 (11.1) | 0 (0.0) |
| Prophylactic antithrombotic agent | 1 (2.0) | 2 (3.9) | 0 (0.0) | 0 (0.0) |
| Thienopyridine | 1 (2.0) | 3 (5.9) | 0 (0.0) | 0 (0.0) |
| ADP receptor antagonist (non-thienopyridine) | 0 (0.0) | 0 (0.0) | 0 (0.0) | 0 (0.0) |
| Cox-2-Inhibitor | 4 (8.2) | 2 (3.9) | 0 (0.0) | 3 (18.8) |
| NSAID/non-cox-2-inhibitor | 15 (30.6) | 16 (31.4) | 4 (22.2) | 5 (31.3) |
| ACEI/ARB/Direct Renin Inhibitor | 31 (63.3) | 38 (74.5) | 10 (55.6) | 10 (62.5) |
| Beta-Blocker | 13 (26.5) | 17 (33.3) | 2 (11.1) | 2 (12.5) |
| **Medications taken within 24 hr before surgery, n (%)** |  |  |  |  |
| Non-study Statin | 22 (44.9) | 17 (33.3) | 0 (0.0) | 0 (0.0) |
| Non-study Antifibrinolytic agent | 1 (2.0) | 0 (0.0) | 0 (0.0) | 0 (0.0) |
| Therapeutic dose warfarin | 0 (0.0) | 0 (0.0) | 0 (0.0) | 0 (0.0) |
| Therapeutic dose direct thrombin/factor Xa inhibitor | 0 (0.0) | 0 (0.0) | 0 (0.0) | 0 (0.0) |
| Prophylactic antithrombotic agent | 1 (2.0) | 3 (5.9) | 0 (0.0) | 1 (6.3) |
| Thienopyridine | 0 (0.0) | 1 (2.0) | 0 (0.0) | 0 (0.0) |
| ADP receptor antagonist (non-thienopyridine) | 0 (0.0) | 0 (0.0) | 0 (0.0) | 0 (0.0) |
| Cox-2-Inhibitor | 1 (2.0) | 1 (2.0) | 0 (0.0) | 1 (6.3) |
| NSAID/non-cox-2-inhibitor | 2 (4.1) | 2 (3.9) | 0 (0.0) | 1 (6.3) |
| ACEI/ARB/Direct Renin Inhibitor | 15 (30.6) | 19 (37.3) | 6 (33.3) | 2 (12.5) |
| Beta-Blocker | 12 (24.5) | 17 (33.3) | 2 (11.1) | 2 (12.5) |

TXA, tranexamic acid

* One patient was included in the trial because no chronic statin therapy was reported; only once in the trial it was found out that the patient was already on statin at home and the study drug was discontinued. The patient was included in the intention-to-treat analyses.
