# Supplementary material for: Tranexamic acid and rosuvastatin in patients at risk of cardiovascular events after noncardiac surgery: a pilot of the POISE-3 randomized controlled trial
Source: Pilot Feasibility Stud. 2020 Jul 21;6:104. doi: 10.1186/s40814-020-00643-9 (PMC7372857; doi:10.1186/s40814-020-00643-9)
Supplement: Supplementary file 5 — Additional File 5. Additional Table. Effect of TXA on hemoglobin change from preoperative to post-operative day 1, and from preoperative to the lowest postoperative level. [file 40814_2020_643_MOESM5_ESM.doc]

**Additional Table**. Effect of TXA on hemoglobin change from preoperative to post-operative day 1, and from preoperative to the lowest postoperative level

|  | **TXA (N=49)** | | **Placebo (N=51)** | | **Treatment effect** | |
| --- | --- | --- | --- | --- | --- | --- |
| **Hb change definition** | **Mean hemoglobin change^a^ ± SD, g/L** | **Missing data, n (%)** | **Mean hemoglobin change^a^ ± SD, g/L** | **Missing data, n (%)** | **Mean difference in hemoglobin change between TXA and placebo (95% CI)** | **p value** |
| **From preoperative to day 1 after surgery** | 21.5 ± 11.4 | 7 (14.3) | 21.2 ± 10.4 | 2 (4.1) | 0.29 (-4.24, 4.82) | 0.898 |
| **From preoperative to the lowest value after surgery** | 27.5 ±13.9 | 6 (12.2) | 28.0 ±13.6 | 1 (2.0) | -0.53 (-6.22, 5.15) | 0.852 |

TXA, tranexamic acid. SD, standard deviation

^a^ Change was calculated for each patient as preoperative hemoglobin minus postoperative hemoglobin
